# Hyperglycemia, diabetes, and de novo diabetes in patients hospitalized in intensive care units for COVID-19 in Colombia: Results from a longitudinal cohort study

**DOI:** 10.2478/jccm-2025-0026

**Published:** 2025-07-31

**Authors:** John Jaime Sprockel, Ana Maria Perez, Maria Camila Chamorro, Jose Alejandro Vergel, Ximena Espinosa, Juan Carlos Vargas, Carlos Angarita, Jhon Edinson Parra

**Affiliations:** El Tunal Hospital, Bogota, Cundinamarca, Colombia; Fundación Universitaria de Ciencias de la Salud, Bogota, Colombia

**Keywords:** COVID-19, newly diagnosed diabetes, diabetes mellitus, intensive care, mortality, organic failure

## Abstract

**Introduction:**

Hyperglycemia and diabetes have been identified as risk factors for severe COVID-19 and death, with a high rate of reported de novo diabetes. We evaluated their incidence and relationship with adverse outcomes in critically ill COVID-19 patients.

**Methods:**

Prospective single-center longitudinal cohort study in adults hospitalized in intensive care units for confirmed COVID-19. ROC curves for serum glucose and glycated hemoglobin were plotted in relation to 60-day mortality. A Cox proportional hazards model was used to assess the association of diabetes and de novo diabetes with 60-day mortality.

**Results:**

547 patients were included, with a mean age of 59.8 years; 133 (24.3%) had a history of diabetes, and 67 (12.2%) had de novo diabetes. At 60 days, 317 (57.9%) had died. For mortality, the AUC for glucose at admission was 0.55 (95% CI: 0.48 – 0.62) and 0.51 (95% CI: 0.41 – 0.62) for glycated hemoglobin. In the Cox model, diabetes had an HR of 0.888 (95% CI: 0.695 – 1.135, p: 0.344), history of DM had an HR of 0.881 (95% CI: 0.668 – 1.163, p: 0.371), and de novo diabetes had an HR of 0.963 (95% CI: 0.672 – 1.378, p: 0.835).

**Conclusion:**

There was a high incidence of de novo diabetes in patients hospitalized in intensive care for COVID-19. Neither hyperglycemia, history of diabetes, nor de novo diabetes were associated with the development of complications or 60-day mortality.

## Introduction

Diabetes Mellitus (DM) has been identified as a precipitating factor for organ dysfunction and mortality in patients with COVID-19 [1,2,3]. Studies have suggested that this condition is associated with chronic endothelial dysfunction, predisposing individuals to severe infections by altering the glycocalyx and endothelial cells, increasing the adhesion of inflammatory cells, and promoting a procoagulant and antifibrinolytic state in the vascular system [4]. Additionally, the binding of the SARS-CoV-2 virus to the angiotensin-converting enzyme 2 (ACE2) receptor in pancreatic islets could lead to the destruction of these cells [5], resulting in the development of de novo DM in predisposed individuals [6, 7], or inadequate blood glucose control, exacerbating preexisting disease [8].

Other studies have documented elevated levels of amylase and lipase, as well as changes in the pancreatic duct, suggesting a direct impact of the virus on the pancreas [9]. SARS-CoV-2 binds to specific receptors such as transmembrane serine protease 2 (TMPRSS2), ACE2, and dipeptidyl peptidase 4 (DPP-4) present in pancreatic beta cells. Autopsy studies and organoid models have confirmed the infiltration and replication of the virus in these cells. This infection disrupts beta cell function, reducing insulin production and increasing glucagon and trypsin levels. Some beta cells become “hormonally negative.” These changes may contribute to the alterations in blood glucose levels observed in COVID-19 patients and potentially to the development of new-onset diabetes [9].

The use of glucocorticoids in the treatment of moderate to severe COVID-19 infection cannot be overlooked, as they act as an additional factor contributing to hyperglycemia [10]. Indirectly, isolation measures during the COVID-19 pandemic have led to loneliness, depression, reduced physical activity, and unhealthy eating patterns. Together, these factors significantly increase the risk of developing Diabetes Mellitus, which is reflected in COVID-19 patients as a more significant metabolic burden, including the development of dyslipidemia, hypertension, obesity, atherosclerotic disease, and chronic kidney disease [11].

In this way, hyperglycemia, type 2 DM, and *de novo* diabetes present particular characteristics during the COVID-19 pandemic that have not yet been explored in our setting. Therefore, the central objective of this study is to analyze the relationship between hyperglycemia and diabetes with the development of complications and death, and specifically, to study the incidence and relationship of *de novo* diabetes with adverse outcomes in a population of critically ill patients hospitalized for severe COVID-19.

## Materials and Methods

A prospective longitudinal cohort study was conducted at a single center, including patients hospitalized in the intensive care unit of the Unidad de Servicios de Salud – Hospital El Tunal, part of the Subred de Servicios de Salud del Sur de Bogotá, Colombia, between May 2020 and April 2022. Inclusion criteria were individuals over 18 years of age hospitalized in the intensive care unit with a diagnosis of severe COVID-19 (defined as ICU admission due to risk of respiratory failure or need for orotracheal intubation, septic shock, or multi-organ dysfunction) confirmed by RT-PCR for SARS-CoV-2 [12]. Patients who died within the first 24 hours of hospital stay, those with therapeutic dissent, pregnant patients, and those without an adequate medical history record were excluded.

Daily censuses of hospitalized patients in various units were reviewed to identify cases meeting the inclusion criteria. A virtual form was then completed, considering the variables recommended by the International Severe Acute Respiratory and Emerging Infections Consortium (ISARIC) of the World Health Organization.

The presence of type 2 diabetes mellitus was defined based on the report of this condition in the admission medical history or subsequent evaluations. The diagnosis of de novo diabetes mellitus was assigned to cases where multiple random glucometer readings showed levels above 200 mg/dl, along with elevated glycated hemoglobin (HbA1c) levels of 6.5% or higher [13].

A descriptive analysis of the population was conducted, recording absolute frequencies and percentages for qualitative variables, and measures of central tendency and dispersion according to their distribution for quantitative variables. ROC curves were constructed for the different levels of glucose and HbA1c at admission with respect to the 60-day mortality outcome, calculating the area under the curve (AUC) with their respective confidence intervals. A series of bivariate analyses were then performed using the Cox proportional hazards model, reporting Hazard Ratios (HR) and p-values for the association of type 2 diabetes mellitus or de novo diabetes with the 60-day mortality outcome. A multivariate analysis was planned if the p-value achieved was less than 0.1.

Kaplan-Meier curves were constructed to analyze the association of type 2 diabetes mellitus or de novo diabetes with the 60-day mortality outcome, obtaining the HR value with its confidence interval and the p-value using the LogRank test.

The study was approved by the Ethics and Research Committee of the Fundación Universitaria de Ciencias de la Salud (Act number 656 of 2022) and the Subred de Servicios de Salud del Sur (Act number 138 of 2020). It was determined that obtaining informed consent was not necessary.

## Results

**Fig. 1.**
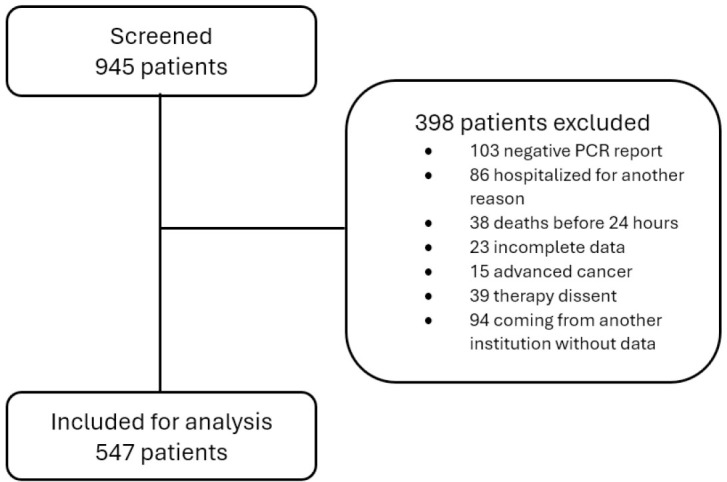
Patient flowchart

Figure 1 shows the flowchart of the patients indicating how during the research period the final inclusion of 547 patients in the database for analysis was achieved. The general characteristics of the population are described in Table 1. The average age was 59.8 years, 196 (36.0%) patients were women, and the average duration of symptoms before ICU admission was 8 days. Hypertension was present in 237 (43.3%) patients, obesity in 154 (28.1%), smoking in 131 (23.9%), and chronic obstructive pulmonary disease in 107 (19.5%). The average lymphocyte count was 982 cells/μL, C-reactive protein was 18.3 mg/dL, ferritin was 1054 ng/mL, D-dimer was 220 μg/mL, LDH was 1163 U/L, and 47% of the total population had positive troponin I results (Table 2). 529 patients (96.7%) received corticosteroid therapy, predominantly dexamethasone at a dose of 6 mg per day, following the dosing recommendations established by the RECOVERY trial.

**Table 1.** General characteristics of the population

**Characteristics**	**Total (n=547)**	**No diabetes (n= 347)**	**Type 2 diabetes (n= 133)**	**De novo diabetes (n= 67)**
Female sex, n (%)	196 (36%)	130 (37.5%)	62 (46.7%)	25 (37.3%)
Age (years), mean (SD)	59.8 (14.3)	59.83 (17.0)	60.2 (13.9)	59.6 (14.2)
20–29, n (%)	19 (3.4%)	17 (4.8%)	3 (2.3%)	3 (4.5%)
30–39	36 (6.5%)	29 (8.3%)	7 (5.2%)	3 (4.5%)
40–49	76 (13.9%)	65 (18.7%)	9 (6.8%)	7 (10.5%)
50–59	112 (20.4%)	84 (24.2%)	25 (18.8%)	19 (28.3%)
60–69	154 (28.1%)	103 (29.6%)	49 (36.8%)	26 (39.0%)
70–79	112 (20.4%)	77 (22.2%)	31 (23.3%)	15 (22.4%)
80–89	37 (6.7%)	28 (8.06%)	9 (6.8%)	4 (6.0%)
<=90	0 (0%)	0 (0%)	0 (0%)	0 (0%)
Obesity, n (%)	154 (28.1%)	112 (32.3%)	41 (30.8%)	20 (30.0%)

**Comorbidities, n (%)**				
Hypertension	237 (43.3%)	140 (40.3%)	93 (70.0%)	28 (42.0%)
Chronic Heart Disease (excluding Hypertension)	60 (19.9%)	38 (11.0%)	22 (16.5%)	8 (12.0%)
Chronic kidney disease	22 (4.0%)	11 (3.2%)	11 (8.3%)	1 (1.5%)
Smoking	131 (23.9%)	99 (28.5%)	31 (23.3%)	13 (19.4%)
Chronic lung disease	107 (19.5%)	72 (20.7%)	34 (25.6%)	14 (21.0%)
Chronic Neurological Disease or Dementia	25 (4.5%)	16 (4.6%)	9 (6.8%)	3 (4.5%)
Human Immunodeficiency Virus	1 (0.18%)	1 (0.3%)	0 (0%)	0 (0%)
Autoimmune disease	6 (1.09%)	6 (1.7%)	0 (0%)	0 (0%)
Cancer	15 (2.74%)	9 (2.6%)	6 (4.5%)	3 (4.5%)
Chronic liver disease	6 (1.09%)	5 (1.5%)	1 (0.8%)	1 (1.5%)
Symptom duration before ICU (days), mean (SD)	8 (4.23)	8 (4.23)	8 (4.13)	8 (4.23)

SD: Standard Deviation

**Table 2.** Laboratory Results and Outcomes

**Characteristics**	**Total (n=547)**	**No diabetes (n= 347)**	**Type 2 diabetes (n= 133)**	**De novo diabetes (n= 67)**
**Laboratories, mean (SD)**				
White blood cell count (×10^3^ cells/μL)	11894 (9607)	11894 (9607)	11911 (9685)	11870 (9670)
Lymphocytes (×10^3^ cells/μL)	982 (1626)	905 (772)	867 (724)	899 (785)
Platelet count (×10^3^ cells/μL)	241723 (95848)	241723 (95848)	242059 (95948)	241986 (96157)
Lactate (mmol/L)	1.96 (1.78)	1.96 (1.78)	1.98 (1.79)	1.96 (1.79)
Creatinin (mg/dL)	0.28 (0.73)	0.27 (0.73)	0.29 (0.74)	2.0 (0.74)
Aspartate aminotransferase (U/L)	126 (424.8)	126 (424.8)	127 (429.12)	126 (428.4)
Alanine aminotransferase (U/L)	97 (235.62)	97 (235.62)	98 (237.91)	93 (208.2)
Prothrombin time (seg)	12 (9.13)	12 (9.13)	12 (9.22)	12 (9.21)
Partial thromboplastin time (seg)	28.2 (17.7)	28.2 (17.7)	28.2 (17.92)	28.3 (17.92)
C-reactive protein (mg/L)	18.3 (20.21)	18.3 (20.21)	18.4 (20.36)	18.4 (20.34)
Ferritin (ng/mL)	1054 (635.43)	1054 (635.43)	1058 (628.93)	1052 (635.49)
D – Dimer (μg/mL)	220 (2100)	220 (2100)	224 (2122)	224 (2122)
Lactate dehydrogenase, U/L	1163 (5530)	1163 (5530)	1163 (5582)	1167 (5568)
Positive troponin, n(%)	257 (47.0%)	172 (50.0%)	49 (37.0%)	36 (54.0%)
PaO_2_/FiO_2_ Ratio	103 (61.41)	103 (61.41)	104 (61.77)	103 (61.68)

**Clinical Prediction Rules, mean (SD)**				
Acute Physiology And Chronic Health				
Evaluation (APACHE) II at admission	14.58 (7.16)	14.58 (7.16)	14.62 (7.17)	14.56 (7.13)
Sequential Organ Failure Assessment (SOFA) at admission	5.22 (3.55)	5.22 (3.55)	5.21 (3.57)	5.22 (3.54)
CURB-65	1.82 (1.03)	1.82 (1.03)	1.82 (1.04)	1.81 (1.03)

**Organ Dysfunctions, n (%)**				
Severe ARDS (Pa/FiO_2_ <100)	341 (62.34%)	259 (75.0%)	77 (58.0%)	50 (75.0%)
Acute Liver Injury	60 (10.96%)	53 (15.2%)	7 (5.3%)	11 (16.4%)
Coagulopathy	59 (10.78%)	46 (13.31%)	12 (9.0%)	13 (19.4%)
CNS Involvement	50 (9.14)	37 (11.0%)	12 (9.0%)	12 (18.0%)
Acute Myocardial Injury	141 (25.77)	108 (31.1%)	30 (23.0%)	23 (34.3%)
Pulmonary embolism, n (%)	65 (11.88%)	51 (15.0%)	13 (10.0%)	17 (25.3%)
Length of Hospital Stay, mean (SD)	23.6 (18.45)	23.6 (18.45)	23.75 (18.58)	23.68 (18.56)
Death, n (%)	317 (58.0%)	211 (61.0%)	65 (49.0%)	41 (61.2%)

SD: standard deviation, CNS: central nervous system

60-day mortality occurred in 317 cases (58.0%), with an average hospital stay of 23.6 days (SD 18.4). Severe acute respiratory distress syndrome (ARDS) (PaO2/FiO2 <100 mmHg) was present in 341 patients (62.34%), myocardial injury in 141 patients (25.7%), pulmonary thromboembolism in 65 patients (11.88%), acute liver injury in 60 patients (10.96%), and coagulopathy in 59 patients (10.78%). Regarding severity scales at ICU admission, the average APACHE II score was 14.58 (SD 7.16), SOFA was 5.22 (SD 3.55), and CURB 65 was 1.82 points (SD 1.03) (Table 2).

Of the total population, 67 patients met the established criteria for de novo type 2 diabetes mellitus, of which 37.3% were female. The average age was 59.6 years, with the highest incidence in the 60–69 age range (39%), followed by the 50–59 age range (28.3%). Compared to other diabetic and non-diabetic populations, the majority of patients fell within these age ranges. Obesity was present in 20 patients (30.0%) in this group. Hypertension was the most common comorbidity with 28 patients (42.0%), followed by chronic obstructive pulmonary disease (21.0%) and smoking (19.4%). No patients had immunosuppression (HIV) or autoimmune diseases. The median duration of illness before ICU admission was 8 days, which was the same as in the other groups.

According to the laboratory tests, the results obtained in the de novo diabetes mellitus group were very similar compared to the other two groups. However, the percentage of positive troponins in this group was higher, reaching 54.0%. In terms of severity scales, this group scored similarly to the populations with and without diabetes. Based on these results, the most common organ dysfunction in the de novo diabetes group was acute respiratory distress syndrome (ARDS), at 75.0%, followed by acute myocardial injury at 34.3%. The 60-day mortality rate was 61.2%, corresponding to 41 patients.

**Fig. 2.**
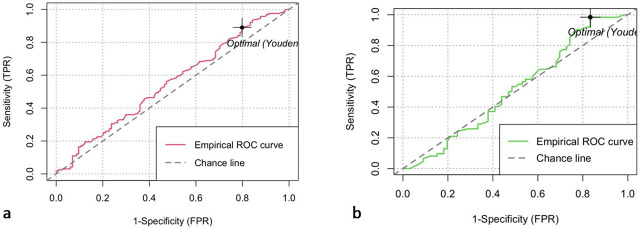
Receiver Operating Characteristic (ROC) Curve for the association between 60-day mortality and blood glucose (a) or glycated hemoglobin (b)

**Fig. 3.**
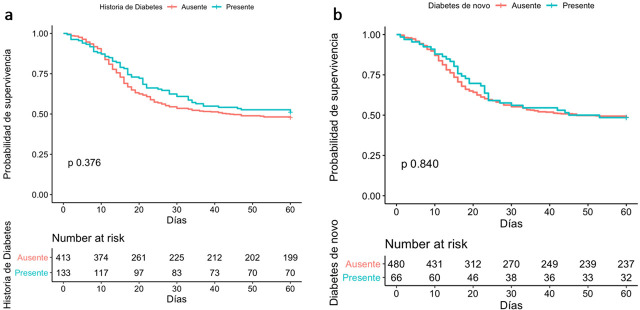
Kaplan-Meier Curve for the association between 60-day mortality and the presence of a history of type 2 diabetes (a) or de novo diabetes (b). The p-value was calculated using the Log Rank Test technique

Figure 2 presents the receiver operating characteristic curve for the association of blood glucose (1a) and glycated hemoglobin (1b) with mortality. An area under the ROC curve of 0.55 (95% CI: 0.48 – 0.62) was found for serum glucose and 0.51 (95% CI: 0.41–0.61) for glycated hemoglobin, ruling out their association with this outcome. Table 3 shows the results of the bivariate analyses. The first part shows the Cox proportional hazards analysis for 60-day mortality in relation to all cases of diabetes mellitus, with an HR of 0.888 (95% CI: 0.69 to 1.13, p=0.344). For the history of type 2 DM, the HR was 0.881 (95% CI: 0.66 to 1.16, p=0.371) and for de novo diabetes, the HR was 0.962 (95% CI: 0.67 to 1.37, p=0.835). Figure 3 shows the Kaplan-Meier curves obtained for the association of the presence of type 2 diabetes (2a) and de novo diabetes with 60-day mortality, with p-values from the Log Rank test of 0.376 and 0.840, respectively.

**Table 3.** Results of Bivariate Analyses

	**HR**	**95% Confidence Interval**	**p value**
All diabetes	0.888	0.6953 – 1.135	0.344
History of diabetes	0.881	0.6675 – 1.163	0.371
*De novo* diabetes	0.963	0.672 – 1.378	0.835

	**OR**	**95% Confidence Interval**	**p value**
Acute kidney injury	0.997	0.874 – 1.137	0.963
Liver injury	1.012	0.932 – 1.099	0.776
Acute myocardial injury	1.034	0.922 – 1.160	0.558
Venous thromboembolism	1.115	1.023 – 1.214	0.011

The second part of Table 3 shows the results of the bivariate analyses using simple logistic regression for the development of acute myocardial injury, renal injury, and hepatic injury, without finding any association with the diagnosis of de novo diabetes. An OR of 1.11 with a 95% CI of 1.02 to 1.21 and a p-value of 0.011 was documented for the association of de novo diabetes with the occurrence of acute pulmonary thromboembolism.

## Discussion

COVID-19 has posed an unprecedented challenge in the field of healthcare, causing millions of deaths and adverse outcomes worldwide. Despite extensive exploration from various approaches regarding the impact of hyperglycemia, diabetes mellitus, and *de novo* diabetes in patients affected by severe COVID-19, there remains considerable fragmentation concerning the influence of these conditions [14], with notable variations in the literature from different regions of the world [15]. In this extensive Latin American cohort of critically ill patients, the high prevalence of diabetes in patients with severe COVID-19 is confirmed, including a significant proportion of newly diagnosed cases. Within the framework of this study, no statistically significant association could be established between hyperglycemia, diabetes mellitus, and *de novo* diabetes, and the various outcomes, including mortality and complications such as multiple organ dysfunction.

We found an area under the ROC curve for the association of hyperglycemia with 60-day mortality of 0.55 (95% CI: 0.48–0.62). The work carried out by Xiao et al. at the hospital in Changsha, China, showed that hyperglycemia is an important factor for predicting severe cases, demonstrating an area under the ROC curve of 0.84 (95% CI: 0.78–0.90, p <0.001) [16]. On the other hand, in a retrospective cohort conducted by Chang et al. at a tertiary hospital in South Korea, an area under the ROC curve for fasting blood glucose to predict mortality of 0.76 (95% CI: 0.66–0.84, p <0.0001) was shown [17].

Additionally, in our study, an area under the ROC curve for the association of HbA1c with 60-day mortality of 0.51 (95% CI: 0.41–0.61) was found, ruling out its association with this outcome. Similar to our results, Greco et al. in a population from Modena, Italy, reported an area under the ROC curve for the association between HbA1c and mortality of 0.19 without statistical significance, when studying the prevalence and possible complications in patients with COVID-19 [18]. In contrast, in the study conducted by Liu et al. at Tongji Hospital in Wuhan, China, which aimed to evaluate the prognosis of DM and COVID-19 patients as well as the impact of prior glycemic control, the ROC curve analysis was used to identify the optimal HbA1c cutoff of 8.6%, with an area under the ROC curve of 0.90 (95% CI: 0.83–0.98, p<0.001), sensitivity of 100%, and specificity of 82%, predicting disease worsening in these patients [19].

In the analyzed population group, 24.0% had a history of type 2 diabetes mellitus, consistent with global data [1, 3]. The median age was 60.2 years, lower than the 73 years reported in the United Kingdom [3], but similar to initial studies in Wuhan, China, of 56 and 62 years [20]. Forty-six percent of the patients were women, and although diabetes was mentioned as a comorbidity in other studies, specific characteristics in this group were not distinguished. The prevalence of obesity in our study (30.8%) was higher than in the United Kingdom (11%) [2]. The main comorbidities included hypertension, chronic lung disease, and smoking, aligning with other studies that highlight hypertension as the most common comorbidity in patients admitted to intensive care units.

Regarding *de novo* diabetes and COVID-19, the analysis of an extensive cohort from the U.S. Department of Veterans Affairs found that after recovering from COVID-19, there is an increased risk of developing diabetes and needing treatment to control blood glucose in the long-term recovery phase [21]. The study revealed that the risk of developing diabetes was 40.0%, translating to 13.46 new cases of diabetes per 1,000 people at 12 months. Notably, this risk increased according to the severity of the acute COVID-19 infection.

In the present study, an incidence of 12.2% (67/547) of *de novo* diabetes was documented, a finding similar to that reported in a retrospective analysis which documented it in 16% of cases (26/166) of COVID-19, where no significant increase (OR 2.61; 95% CI, 0.86–7.88; p = 0.090) in the risk of composite outcomes (mechanical ventilation, ICU admission, and death) was observed in the group with diabetes (both newly diagnosed and pre-existing) [22]. On the other hand, a study that included patients hospitalized for COVID-19 found that 21% (94/453) of patients had *de novo* diabetes, reporting a significant increase in all-cause mortality at 30 days (HR 9.42; 95% CI, 2.18–40.7) in a multivariable analysis [23]. An Italian cohort of 413 hospitalized patients found 5% of cases (21/413) of de novo diabetes with a significant increase in severe COVID-19, defined as ICU admission and death (RR 3.06; 95% CI, 2.04–4.57) [24].

A random-effects meta-analysis that included 8 studies with 4,270,747 COVID-19 patients and 43,203,759 controls found that COVID-19 was associated with a 66% higher risk of developing new-onset diabetes. This means that individuals who had COVID-19 have a significantly higher likelihood of developing diabetes after recovering from SARS-CoV-2 infection [25].

It is worth noting that the nearly universal use of corticosteroids in this population (96.7%), predominantly dexamethasone at a dose of 6 mg/day as per RECOVERY trial guidelines, may have influenced the high incidence of de novo diabetes. However, due to the lack of variability in corticosteroid exposure among participants, we were unable to assess its potential role as a contributing factor to new-onset diabetes in this cohort.

Mortality in our study population with a history of diabetes was lower compared to groups of patients without a history of diabetes or with *de novo* diabetes; however, it remains high compared to populations in countries like Italy and the United States, which could be related to differences in healthcare systems in each country (2). On the other hand, Vargas-Vásquez et al. analyzed 317 hospitalized COVID-19 cases in a reference center in Mexico City, Mexico, reporting that not only was previously known DM a risk factor for negative outcomes in severe COVID-19 infection such as mortality, but also undiagnosed DM and prediabetes acted as risk factors for worse outcomes, with OR: 5.76 (95% CI: 1.46 to 27.1) and OR: 4.15 (95% CI: 1.29 to 16.75), respectively [13]. Given this, the main finding of the present study is that patients with a prior history of DM during ICU stay for COVID-19 infection did not have a statistically significant association with 60-day mortality.

The current study has several limitations, primarily the lack of information on blood glucose and HbA1c levels for all patients, which could be related to the previously presented results. Additionally, data were collected exclusively at a single center, raising concerns about the generalizability of the results to different populations.

Among the strengths, we highlight that it is a large cohort of critically ill patients throughout the four waves of the COVID-19 pandemic. Various types of statistical analyses were conducted, including the relationship of hyperglycemia, which included glycated hemoglobin, as well as multivariate models with a longitudinal component with follow-up up to 60 days after admission. Although it is described as single-center, the hospital became a reference center for critical cases during the pandemic, increasing from 25 beds to a maximum of 130 during the most critical periods of the first and second waves, representing an area of influence of approximately 2,000,000 inhabitants in the southern part of Bogotá.

## Conclusions

In a Latin American cohort of patients hospitalized in intensive care for severe COVID-19, a high proportion of cases with type 2 diabetes was documented, among which there was a high incidence of *de novo* diabetes. Neither hyperglycemia, history of diabetes, nor *de novo* diabetes were associated with the development of complications or 60-day mortality.
